# Isolated Ipsilateral Hemiparesis From a Pyramidal Decussation Infarct: A Rare Case Confirmed by Magnetic Resonance Imaging

**DOI:** 10.7759/cureus.97461

**Published:** 2025-11-21

**Authors:** Ali H Thahab, Sumeet Bhardwaj, Maher Asfour, Belal Asfour, Akshay Shah

**Affiliations:** 1 College of Medicine: Neurology, Kansas City University of Medicine and Biosciences, Kansas City, USA; 2 College of Medicine: Psychiatry, Kansas City University of Medicine and Biosciences, Kansas City, USA; 3 College of Medicine: Neurology, University of Illinois at Chicago, Chicago, USA; 4 Neurology, Clinical Neurophysiology, Good Samaritan Hospital of San Jose, San Jose, USA; 5 Neurology, Clinical Neurophysiology, O’Connor Hospital, San Jose, USA; 6 Neurology, Clinical Neurophysiology, Regional Medical Center, San Jose, USA

**Keywords:** brainstem infarction, cervicomedullary junction, corticospinal tract, ipsilateral hemiparesis, medulla oblongata, medullary infarction, pyramidal decussation, small vessel ischemic disease

## Abstract

A 38-year-old male smoker with chronic hypertension, anxiety secondary to alcohol withdrawal, chronic alcohol use, and a history of noncompliance with prescribed medications presented with 2 weeks of progressive left-sided weakness, numbness, dizziness, and gait instability. He also reported a month-long history of facial drooping that had not been formally evaluated. Brain magnetic resonance imaging revealed a small acute/subacute infarct in the left posterolateral cervicomedullary junction, at the level of the pyramidal decussation, which produced ipsilateral motor and sensory symptoms. This is a rare presentation of a stroke causing purely ipsilateral neurologic deficits and served as an important reminder of neurologic anatomy caudal to the pyramidal decussation.

## Introduction

Although brainstem strokes account for only 10-15% of all ischemic strokes, they often produce disproportionately complex and misleading clinical presentations due to the high density of vital neural pathways within this region [1]. The medulla, in particular, contains the pyramidal decussation where corticospinal fibers cross, as well as multiple sensory and autonomic tracts [2]. Lesions at this level can generate atypical symptom patterns that complicate diagnosis, delay treatment, and heighten morbidity and mortality. A precise understanding of the vascular anatomy and neuroanatomic organization of the cervicomedullary junction is therefore essential for accurate localization and timely management of these uncommon but potentially devastating syndromes.

This case is notable for demonstrating ipsilateral hemiparesis and sensory loss resulting from a focal infarct at the level of the pyramidal decussation, an exceptionally rare localization seldom described in modern neuroimaging literature. Infarcts at or below the decussation challenge the classic “crossed findings” paradigm traditionally used in neuroanatomic localization, since the corticospinal fibers have already crossed midline at this level. Consequently, such lesions can mimic peripheral neuropathy, spinal cord pathology, or even functional neurological disorders, leading to diagnostic uncertainty and potential mismanagement.

Few documented cases have provided detailed clinico-radiologic correlation of infarcts at the pyramidal decussation producing isolated ipsilateral deficits, particularly in the absence of cranial nerve or cerebellar involvement. This report contributes to the existing literature by offering clear MRI characterization of the lesion, correlating anatomic localization with the patient’s unusual presentation, and situating the findings within the historical framework of Opalski syndrome, a rare variant of lateral medullary infarction in which the stroke extends slightly below the pyramidal decussation into the upper cervical spinal cord. Because the affected corticospinal fibers have already crossed, the resulting weakness appears on the same side as the lesion. In addition to ipsilateral hemiparesis, typical features of lateral medullary infarction may include ipsilateral facial sensory loss, limb ataxia, Horner syndrome, and contralateral loss of pain and temperature sensation.

Finally, this case underscores several broader clinical lessons: (1) the importance of considering medullary pathology in atypical unilateral weakness when initial CT and CTA findings are unremarkable; (2) the diagnostic value of diffusion-weighted magnetic resonance imaging in identifying minute infarcts at the cervicomedullary junction; and (3) the vascular susceptibility of this border-zone region between the anterior spinal and vertebral perforator territories. Together, these neuroanatomic, radiologic, and vascular insights emphasize an under-recognized stroke subtype that can sharpen diagnostic reasoning in both emergency and inpatient neurology practice.

To aid interpretation of this case and ensure clarity for readers less familiar with brainstem anatomy, a brief overview of key neuroanatomical, vascular, and imaging concepts is provided below.

The pyramidal decussation is located at the base of the medulla oblongata, the lowest part of the brainstem. It is the point where most of the corticospinal tract fibers cross to the opposite side before descending into the spinal cord. Lesions above this crossing cause weakness on the opposite side of the body, while lesions at or below it cause weakness on the same side since the fibers have already crossed. The cervicomedullary junction refers to the small transitional zone between the lower medulla and the upper cervical spinal cord, where structures from the brain and spinal cord lie very close together.

The corticospinal tract, also known as the pyramidal tract, carries voluntary motor signals from the brain to the body’s muscles. Damage to this tract leads to weakness, reduces fine motor control, and exaggerates reflexes. A lateral medullary infarct, sometimes called Wallenberg syndrome, typically occurs when blood flow to part of the medulla is interrupted. This produces what are known as “crossed findings” - loss of pain and temperature sensation on the same side of the face but on the opposite side of the body - along with dizziness, limb incoordination, swallowing difficulty, and Horner syndrome (drooping eyelid, small pupil, and loss of facial sweating).

The anterior spinal artery supplies the front two-thirds of the medulla and spinal cord, including the pyramids and crossing motor fibers, while small branches of the vertebral arteries provide additional blood flow to the sides of the medulla. These tiny vessels are particularly vulnerable to damage from long-term high blood pressure, which can cause thickening and narrowing of their walls and result in small, deep strokes known as lacunar infarcts. This process, often referred to as small-vessel disease, can also lead to subtle white-matter changes visible on MRI.

On MRI, recent strokes appear as bright areas on diffusion-weighted imaging (DWI) and dark areas on apparent diffusion coefficient (ADC) maps, confirming restricted water movement in injured tissue. T2-weighted and fluid-attenuated inversion recovery (FLAIR) images are used to identify swelling or chronic small-vessel changes. The term clinico-radiologic correlation simply means matching these imaging findings with the patient’s neurological symptoms and exam results to pinpoint the exact site and cause of the lesion.

In summary, understanding these concepts helps explain how a very small stroke at or just below the pyramidal decussation, like in this patient, can cause weakness and sensory loss on the same side of the body without the cranial nerve findings typically seen in higher medullary strokes.

## Case presentation

A 38-year-old male with a medical history of hypertension and alcohol-use disorder with prior withdrawal episodes presented to the emergency department with approximately two weeks of progressive left-sided numbness and weakness, accompanied by dizziness and increasing difficulty with ambulation. His wife reported that he had developed facial drooping and trouble walking about one month before presentation, though these earlier symptoms had not been formally evaluated. The patient denied any recent illness, trauma, or loss of consciousness. He was not compliant with his home medications, which included chlordiazepoxide, losartan, metoprolol succinate, olanzapine, ondansetron as needed, and pantoprazole. He reported smoking half a pack of cigarettes per day and consuming over 750 mL of whiskey daily. Family history was notable for hypertension and type 2 diabetes in his mother, and depression in his maternal uncle.

On arrival, vital signs were notable for a blood pressure of 165/124 mmHg, heart rate of 100 beats per minute, temperature of 36.7 °C, and oxygen saturation of 100% on room air. On neurologic examination, the patient demonstrated reduced finger taps and diminished sensation on the left side. The National Institutes of Health Stroke Scale (NIHSS) score on presentation was an estimated 2, reflecting mild deficits limited to left-sided weakness and sensory loss without cranial nerve or language involvement.

**Figure 1:**
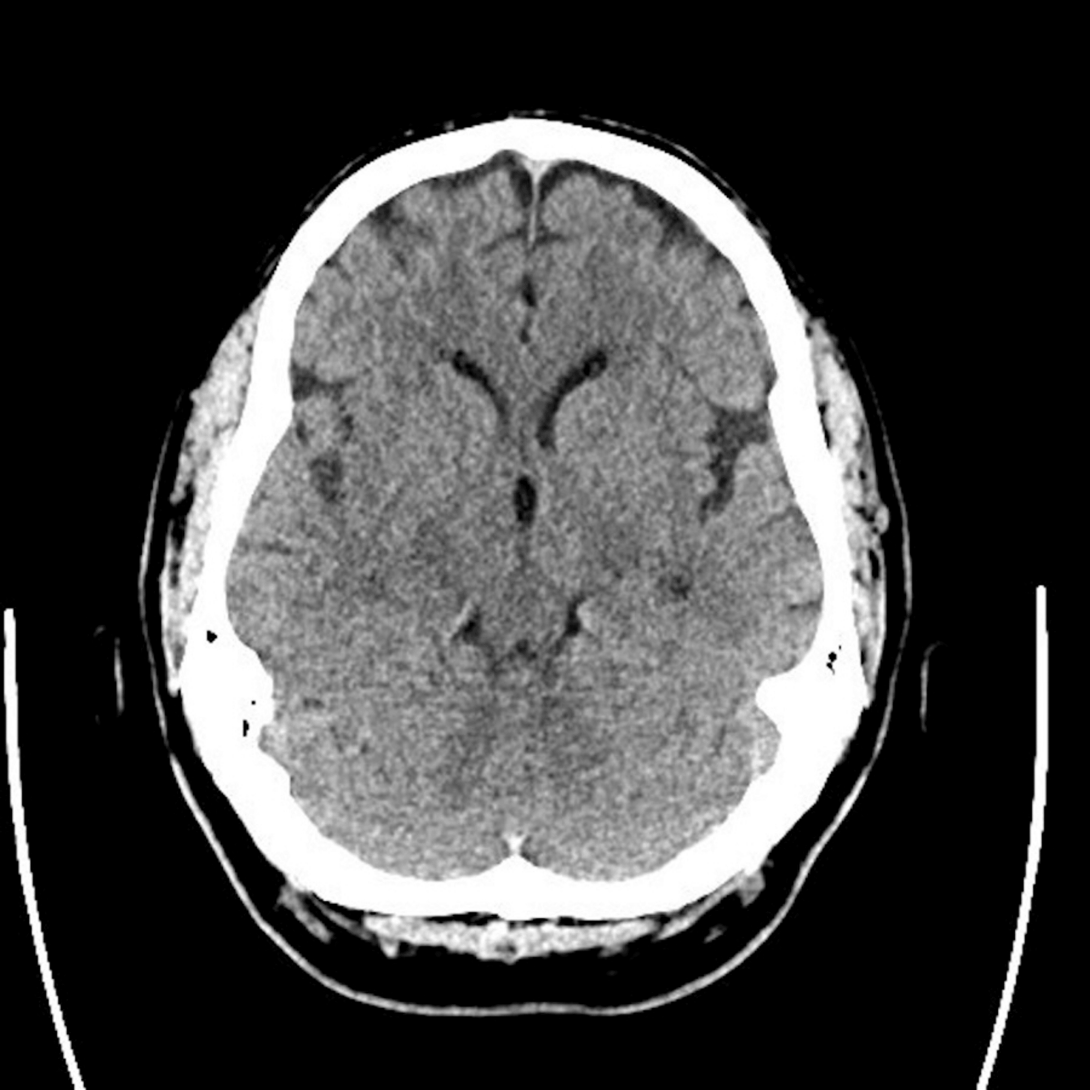
Unremarkable initial CT without contrast evaluation of the brain

**Figure 2:**
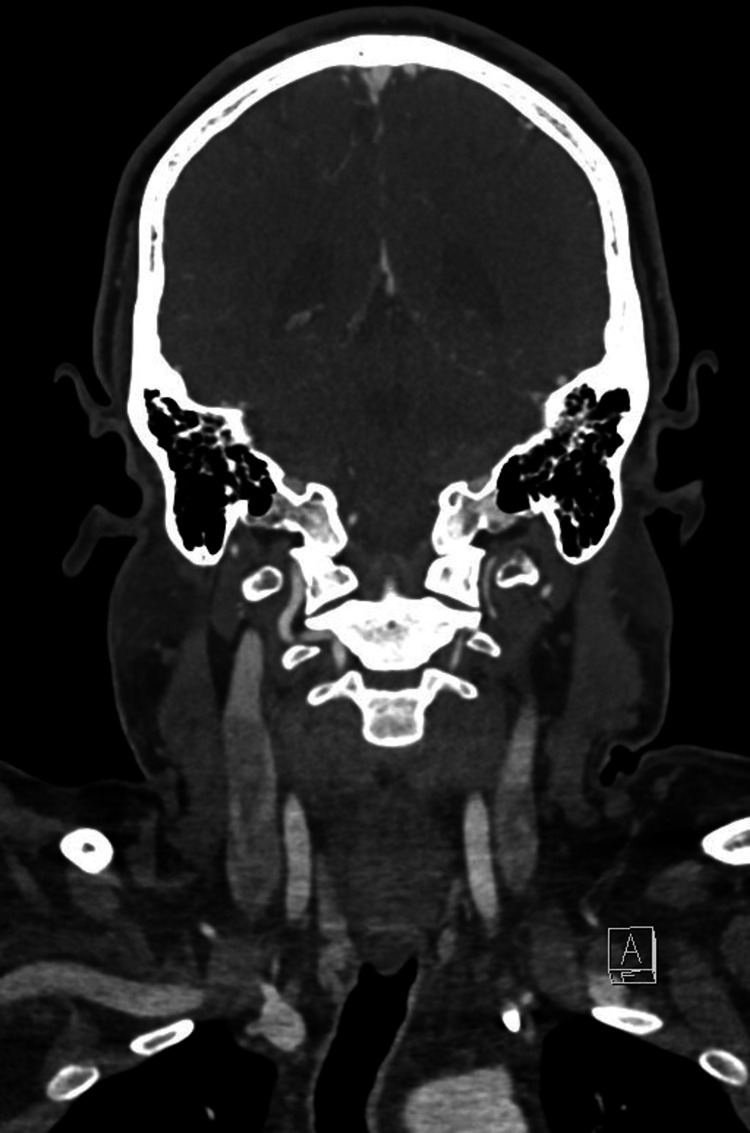
CTA of the head and neck demonstrating patent intracranial arterial vasculature, patent neck arterial vasculature, and no evidence of high-grade stenosis or dissection CTA: computed tomography angiography

There was no facial asymmetry or cranial nerve deficit at the time of examination, and no signs of acute distress. The remainder of the physical exam was unremarkable. Initial non-contrast computed tomography (CT) of the head showed no acute intracranial findings, and computed tomography angiography (CTA) revealed no evidence of large vessel occlusion (Figures 1-2).

The differential diagnosis for unilateral weakness and sensory loss includes cervical spinal cord lesions, peripheral neuropathies, and demyelinating processes. Although a spinal MRI was not obtained in this patient, several clinical features made these alternatives less likely. The absence of lower motor neuron findings, sensory level, or bowel and bladder involvement argued against a spinal cord lesion. Similarly, the lack of asymmetric reflex changes, fasciculations, or distal sensory gradient made peripheral neuropathy improbable. The combination of hemibody involvement without cranial nerve deficits, in conjunction with the focal diffusion restriction at the cervicomedullary junction on brain MRI, supported a central lesion localized to the pyramidal decussation.

The patient was admitted from the emergency department and started on dual antiplatelet therapy. Transthoracic echocardiography (TTE) with a bubble study was performed to assess for a cardioembolic source and demonstrated unremarkable results. His neurological deficits improved rapidly during hospitalization, and given his steady recovery and independence with ambulation, physical and occupational therapy were deferred. He was discharged home with only mild residual numbness in the left lower extremity, and outpatient follow-up was arranged with both his primary care provider and neurology.

Although long-term follow-up data were unavailable, the patient’s early recovery and minimal residual symptoms suggest a favorable prognosis, consistent with prior reports of small, focal infarcts at or near the pyramidal decussation. Most documented cases of cervicomedullary infarction describe substantial functional improvement when vascular risk factors are controlled and secondary prevention strategies such as antiplatelet therapy and blood-pressure management are implemented.

**Figure 3:**
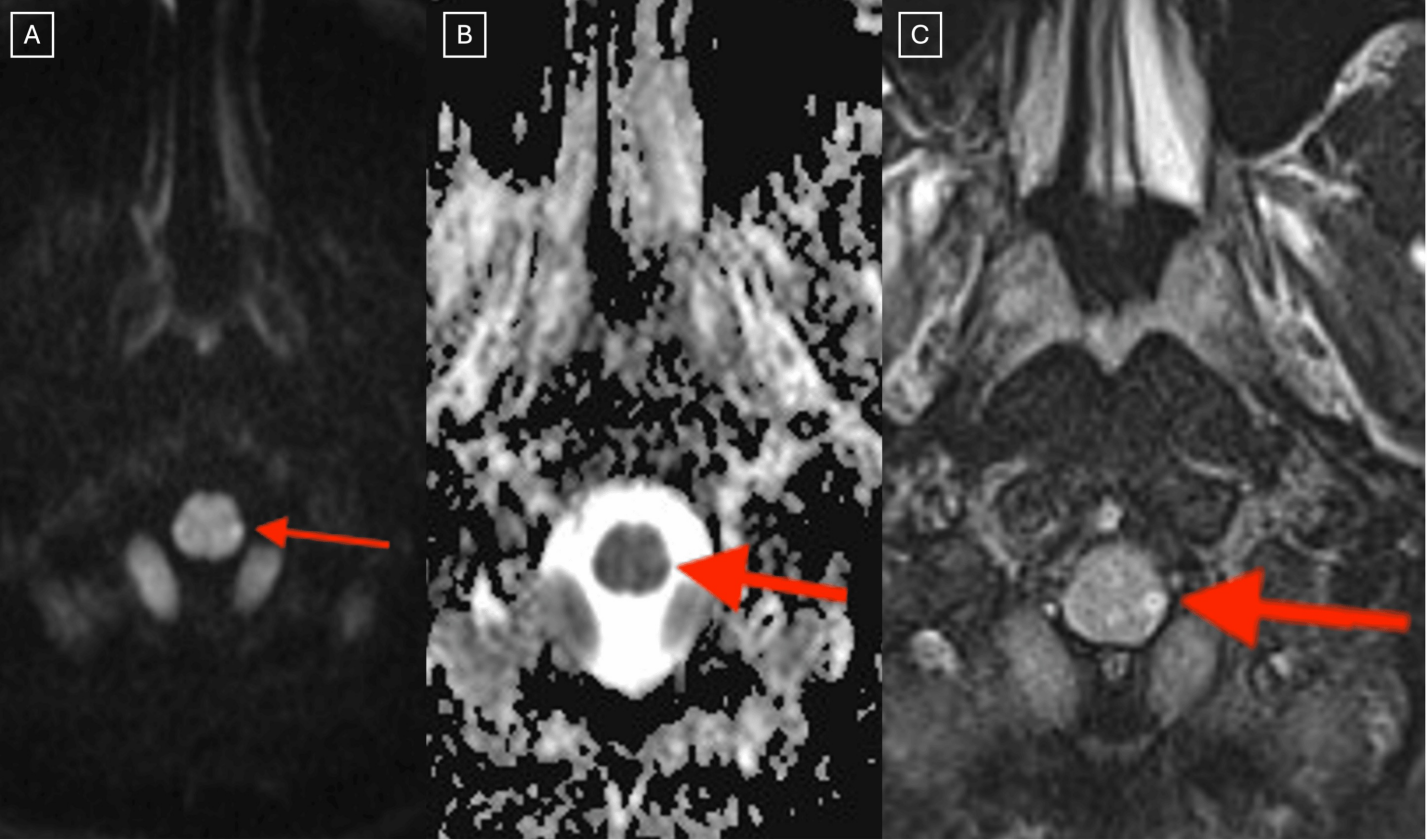
Side-by-side comparison of transverse diffusion-weighted (A), apparent diffusion coefficient (B), and T2-FLAIR (C) MRI of the brain at the level of the pyramidal decussation. The infarct location is indicated by a red arrow. FLAIR: fluid-attenuated inversion recovery

**Figure 4:**
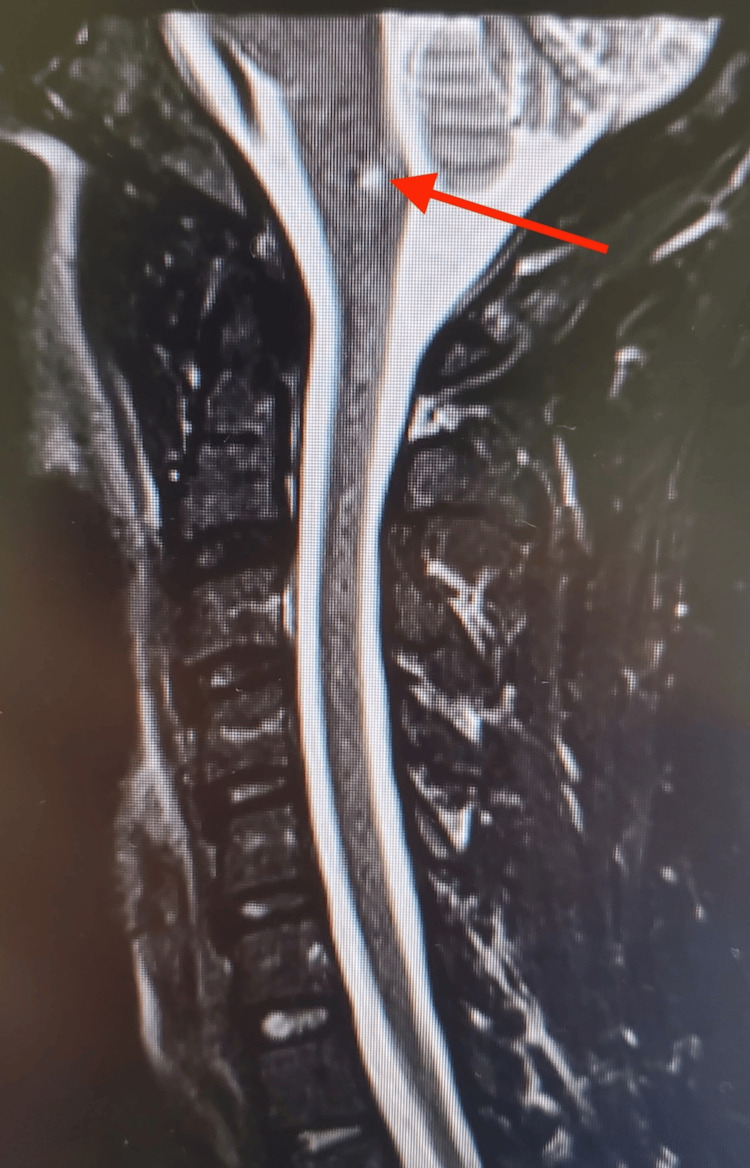
Sagittal MRI image of the brainstem demonstrating a hyperintense region at the site of infarction (red arrow).

Magnetic resonance imaging (MRI) of the brain without contrast was subsequently performed and demonstrated a tiny acute or subacute infarct involving the left posterolateral aspect of the cervicomedullary junction (Figures 3-4).

Notably, this lesion was located caudal to the level of the pyramidal decussation, providing an anatomic explanation for the patient’s ipsilateral (left-sided) motor and sensory symptoms. Additional MRI findings included small T2/FLAIR hyperintense foci in the periventricular and subcortical white matter bilaterally, which were considered nonspecific but possibly related to chronic small vessel ischemic changes. 

## Discussion

This case highlights an exceptionally rare and diagnostically challenging presentation of brainstem stroke - ipsilateral hemiparesis and sensory loss resulting from an infarct at the pyramidal decussation. While most corticospinal tract lesions traditionally cause contralateral weakness, the lesion in this patient produced ipsilateral motor and sensory deficits, which could mimic peripheral or spinal cord disorders. Cases of medullary infarction producing ipsilateral motor and sensory deficits, particularly via the involvement of fibers below the pyramidal decussation, have been observed to be exceedingly rare [3]. This phenomenon has been historically described as Opalski syndrome, a rare variant of Wallenberg syndrome, in which infarction extends caudally into the upper cervical cord or lateral medulla below the decussation [4]. Patients have been reported to develop ipsilateral weakness in addition to the typical features of lateral medullary infarction, such as Horner syndrome or cranial nerve deficits [4]. Only a handful of such cases have been published in the literature [3].

The present case is notable for a lesion confined to the region of the pyramidal decussation, without associated lateral medullary or cranial nerve involvement. This precise localization distinguishes it from classical Opalski or Wallenberg syndromes and represents an uncommon decussation-level infarct scarcely documented in contemporary neuroimaging literature. High-resolution MRI provided a clear clinicoradiologic correlation, demonstrating that lesions localized at or just below the pyramidal decussation can produce isolated ipsilateral hemiparesis in the absence of bulbar signs. This observation underscores a fundamental neuroanatomic principle: the laterality of motor deficits depends on the lesion’s position relative to the corticospinal crossing rather than its broader medullary or cortical territory.

Vascular and pathophysiologic considerations

The complexity and vulnerability of the vascular supply of the cervicomedullary junction predisposed this patient to his highly localized symptoms. The cervicomedullary junction receives blood from both the anterior spinal artery and penetrating branches of the vertebral arteries [1]. The anterior spinal artery, formed by the union of branches from each vertebral artery, supplies the anterior two-thirds of the medulla, including the pyramids, decussating corticospinal fibers, and parts of the medial lemniscus [1]. The penetrating vessels of the vertebral arteries are small end arteries with limited collateral support, which makes them highly susceptible to occlusion from chronic hypertension-related lipohyalinosis or microatheroma formation [5]. Lipohyalinosis, characterized by hyaline and lipid deposition with vessel-wall thickening and foam-cell infiltration, is strongly associated with lacunar infarcts and intracerebral hemorrhage [5,6]. In this case, occlusion of a tiny perforating branch at the pyramidal decussation selectively injured corticospinal fibers that had already crossed, producing ipsilateral hemiparesis without cranial-nerve involvement.

Several additional anatomic and hemodynamic features of the cervicomedullary junction further predispose it to ischemia. The vertebral arteries follow a tortuous course as they ascend around the atlas and axis before joining to form the basilar artery, increasing their susceptibility to mechanical stress and dissection [7]. Although not typically labeled a watershed region, the pyramidal decussation lies at the junction of the anterior spinal artery and vertebral perforator territories - zones vulnerable to ischemia from transient hypoperfusion due to limited collateral flow. Moreover, venous drainage of the medulla is relatively sparse, relying on longitudinal veins with few cross-connections, which may hinder clearance of ischemic metabolites and propagate injury during vascular compromise [8]. These structural vulnerabilities help explain why the cervicomedullary junction is disproportionately affected by subtle vascular insults and why infarcts in this region, though small, produce highly localizing syndromes.

Vascular risk profile and small-vessel disease

The location of infarcts has also been observed to correlate with distinct vascular risk profiles [9]. For example, lacunar infarcts in the deep gray nuclei or internal capsule often occur in younger patients with hyperlipidemia, whereas centrum semiovale infarcts are typically seen in older, hypertensive individuals with multiple lacunes and prominent white-matter hyperintensities [9]. Long-standing hypertension, smoking, alcohol use, and medication noncompliance likely contributed to cerebral small-vessel disease and subsequent ischemia in this patient.

Cerebral small-vessel disease (CSVD) encompasses clinical, imaging, and pathological syndromes affecting the perforating arterioles, capillaries, and venules [6]. It accounts for about one-quarter of ischemic strokes and most hemorrhagic strokes and represents the leading cause of vascular dementia [6]. In early stages, CSVD may be asymptomatic aside from imaging findings, delaying diagnosis and intervention. MRI is the most accurate method for detecting these subtle changes, which include subcortical small infarctions, enlarged perivascular spaces, cerebral microbleeds, and white-matter hyperintensities [6]. In this case, MRI confirmed the acute medullary lesion and revealed chronic microvascular changes, supporting a likely small-vessel etiology.

Clinical and educational implications

Clinically, this case reinforces that ipsilateral hemiparesis does not exclude a central cause. Such presentations can be mistaken for peripheral neuropathy, cervical myelopathy, or functional disorders, particularly when initial CT and CTA are unrevealing. Prompt, targeted MRI of the medulla is essential for detecting small infarcts at the cervicomedullary junction and for establishing accurate localization. From an educational standpoint, this case illustrates the clinical relevance of corticospinal fiber crossing and demonstrates how millimetric variations in lesion position relative to the decussation determine the laterality of motor deficits [10-13].

Although long-term follow-up data were unavailable, the patient’s rapid improvement and discharge with only mild residual numbness suggest a favorable prognosis, consistent with prior reports of small focal infarcts at the pyramidal decussation. Published cases of similar cervicomedullary lesions indicate that most patients experience substantial neurological recovery with appropriate risk-factor management and adherence to secondary prevention therapy [3,4]. Case series and reviews of medullary pyramid infarcts further support that small, strategically located lesions, particularly those sparing the internal capsule and extensive cortical regions, are associated with good functional outcomes [10-15].

Secondary prevention should emphasize strict control of vascular risk factors, including antiplatelet therapy, blood-pressure management, and lifestyle modification, as these measures are critical for reducing recurrence and improving long-term outcomes in patients with small-vessel or brainstem infarcts. Reporting atypical presentations such as this one can improve recognition, shorten diagnostic delays, and enhance understanding of the clinical spectrum of brainstem infarction.

To contextualize the presentation of the current study, Table 1 summarizes previously published cases of ipsilateral hemiparesis due to infarction at or below the pyramidal decussation. Most reported cases have shown either mixed or more extensive medullary lesions with “crossed” findings. In contrast, the present case provides a modern, imaging-supported example of a decussation-level infarct producing isolated ipsilateral deficits, thereby bridging historical descriptions of Opalski syndrome with contemporary neuroimaging evidence.

**Table 1:** Comprehensive summary of all reported cases of ipsilateral hemiparesis resulting from brainstem infarction First Author, Year: Name of the first author and year of publication of the case report or series. Age/Sex: Age and sex of the patient(s) described in the report. Stroke Type/Mechanism: Classification of the stroke (ischemic, hemorrhagic, dissection, etc.) and the underlying mechanism if specified (e.g., vertebral artery dissection, atherosclerosis). Lesion Location: Specific neuroanatomic site of the infarct as identified by imaging (e.g., lower medulla, cervicomedullary junction, paramedian pons, corona radiata). Clinical Presentation: Key neurological findings, including laterality and type of motor/sensory deficits, cranial nerve involvement, and associated symptoms (e.g., hemiparesis, ataxia, Horner syndrome). Imaging Modality: Type of neuroimaging used for diagnosis (e.g., MRI, DWI, MRA). Key Findings: Notable clinical or radiologic observations, such as involvement of decussated corticospinal tract, functional reorganization, or unique syndrome features. LMI: lateral medullary Infarction; IH: ipsilateral hemiparesis; DTI: diffusion tensor imaging; CST: corticospinal tract; MRA: magnetic resonance angiography; VA: vertebral artery

First Author, Year	Age/Sex	Stroke Type/Mechanism	Lesion Location	Clinical Presentation	Imaging Modality	Key Findings	References
Uemura, 2016	7 pts, mixed	Ischemic (LMI)	Lower medulla, cervicomedullary junction	Ipsilateral hemiparesis, ± superficial sensory loss, ± limb ataxia	MRI	IH due to post-decussating pyramidal tract damage	[15]
Li, 2014	1 pt, not stated	Ischemic	Lateral lower medulla	Ipsilateral hemiparesis, lemniscal sensation loss, hypoglossal palsy	MRI, DTI	Decussated CST involvement, a variant of LMI	[16]
Saada, 2014	2 pts, mixed	Ischemic/hemorrhagic	Brainstem, variable	Ipsilateral hemiparesis, prior contralateral stroke in most	MRI	Uncrossed CST or adaptive compensation	[17]
Aaron, 2020	2 pts, not stated	Ischemic	Medullary pyramid	Pure ipsilateral motor hemiparesis	MRI	Isolated medullary pyramid infarct, missed on CT	[18]
Tan, 2019	22 pts, mixed	Ischemic	CST (various levels)	Ipsilateral limb paralysis, mild deficits, and central facioplegia in 1	MRI	Most had a prior contralateral stroke; some had their first stroke due to uncrossed CST	[19]
Ropper, 1979	1 pt, not stated	Ischemic	Right medullary pyramid	Severe hemiplegia sparing face, minimal sensory loss	MRI	Pure motor hemiplegia, good recovery	[10]
Kataoka, 1997	49 pts, mixed	Ischemic	Paramedian pons	Hemiparesis (upper extremity dominant), dysarthria, sensory disturbance	MRI	Faciobrachial dominant hemiparesis, prognosis by lesion level	[20]
Porto, 2009	1 pt, 40/M	Ischemic (VA dissection)	Right hemimedulla	Ipsilateral hemiplegia, hemimedullary syndrome	MRI/MRA	Ipsilateral motor deficit, VA dissection	[21]
Sameshima, 2014	1 pt, 32/M	Ischemic (LMI, VA stenosis)	Caudal medulla, dorsomedial extension	Ipsilateral mild hemiparesis, sensorimotor deficits, Horner, ataxia, dysarthria	MRI/MRA	The lesion likely involved decussating CST and lemniscal fibers	[3]
Wilkins, 2012	1 pt, 52/M	Ischemic	Pyramidal decussation	Quadriplegia, anarthria, sparing face/sensory	MRI	Infarct at the pyramidal decussation	[22]
Inatomi, 2017	14 pts, mean 71 yrs	Ischemic	Frontal cortex, corona radiata, internal capsule, pons	Ipsilateral hemiparesis, most with prior contralateral stroke	MRI, fMRI, TMS	Functional reorganization, uncrossed CST	[23]

Table 2 summarizes previously reported cases of ipsilateral hemiparesis resulting from infarction at or just below the pyramidal decussation. To ensure clinical and anatomical accuracy, only cases with radiologic or pathologic confirmation of lesion level were included. See also Table 1 for a comprehensive summary of all reported cases of ipsilateral hemiparesis secondary to brainstem infarction.

**Table 2:** Summary of reported cases of ipsilateral hemiparesis due to infarction at or just below the pyramidal decussation with radiologic or pathologic confirmation of lesion level This table summarizes all reported cases of ipsilateral hemiparesis due to infarction at or just below the pyramidal decussation, including only those with radiologic (MRI, DTI) or pathologic confirmation of lesion level. Each row details the first author and year, patient demographics, clinical presentation, confirmed lesion location, imaging or pathology modality, presumed etiology, clinical outcome, and relevant medical literature reference. Only cases with direct evidence of lesion at the medullary pyramid or decussation are included to ensure anatomical specificity and clinical relevance. DTI: diffusion tensor imaging; CST: corticospinal tract

First Author, Year	Age/Sex	Clinical Presentation	Lesion Location (Confirmed)	Imaging/Pathology	Etiology	Outcome	References
Ropper, 1979	60/M	Right hemiplegia, face sparing	Right medullary pyramid, ~1 cm below pontomedullary junction	Autopsy	Vascular	Good recovery	[10]
Aaron, 2020 (Case 1)	55/M	Left pure motor hemiparesis	Left medullary pyramid	MRI	Vascular	Good recovery	[18]
Aaron, 2020 (Case 2)	62/F	Right pure motor hemiparesis	Right medullary pyramid	MRI	Vascular	Good recovery	[18]
Wilkins, 2012	52/M	Quadriplegia, anarthria, face sparing	Pyramidal decussation (lower medulla)	MRI	Vascular	Not specified	[22]
Porto, 2009	41/M	Right hemiplegia, hemimedullary syndrome	Right hemimedulla, below pyramidal decussation	MRI	Vertebral artery dissection	Partial recovery	[21]
Uemura, 2016 (4 cases)	Various	Ipsilateral hemiparesis in lateral medullary infarction	Lower medulla, extending to cervico-medullary junction	MRI	Vascular	Not specified	[15]
Li, 2014	60/M	Ipsilateral hemiparesis, lemniscal loss, hypoglossal palsy	Lateral lower medulla (tractography confirmed CST involvement)	MRI, DTI	Vascular	Not specified	[16]

Limitations

Although additional MRI sequences such as gradient echo sequences (GRE) or susceptibility weighted imaging (SWI) were not available, the diffusion-weighted and apparent diffusion coefficient (ADC) images demonstrated clear restricted diffusion without signal loss or blooming, effectively excluding hemorrhage. The lesion pattern and clinical course were consistent with a small ischemic medullary infarct rather than a hemorrhagic event.

## Conclusions

This case highlights the importance of considering brainstem pathology, particularly infarction at the level of the pyramidal decussation, in patients who present with unusual unilateral motor or sensory deficits. Such cases can easily mimic peripheral or spinal disorders, especially when early imaging studies appear normal. Accurate neurological localization and timely MRI are therefore essential for correct diagnosis, as MRI provides the best means of identifying small medullary lesions and correlating them with clinical findings.

Early recognition of these rare stroke patterns allows for prompt initiation of secondary prevention measures, including vascular risk factor optimization, antiplatelet therapy, and patient education to minimize recurrence risk. Greater awareness and detailed reporting of atypical brainstem infarctions will help refine diagnostic accuracy, shorten treatment delays, and expand our understanding of their diverse clinical presentations.

## References

[1] Gowda SN, De Jesus O (2024). Brainstem stroke. StatPearls [Internet].

[2] Lohia A, McKenzie J (2023). Neuroanatomy, pyramidal tract lesions. StatPearls [Internet].

[3] Sameshima T, Morita A, Yamaoka Y, Ichikawa Y (2014). Ipsilateral sensorimotor deficits in lateral medullary infarction: a case report. J Stroke Cerebrovasc Dis.

[4] Opalski A (1946). Un nouveau syndrome sous-bulbaire: syndrome partiel de l’artère vertébro-spinale postérieure [Article in French]. Paris Méd.

[5] Markus HS, Joutel A (2025). The pathogenesis of cerebral small vessel disease and vascular cognitive impairment. Physiol Rev.

[6] Wang Z, Chen Q, Chen J, Yang N, Zheng K (2021). Risk factors of cerebral small vessel disease. A systematic review and meta-analysis. Medicine (Baltimore).

[7] Cacciola F, Phalke U, Goel A (2004). Vertebral artery in relationship to C1-C2 vertebrae: an anatomical study. Neurol India.

[8] Ota T (2023). Functional cerebral venous anatomy: a perspective on venous collaterals. Part II, infratentorial venous system. Stroke Vasc Interv Neurol.

[9] Rutten-Jacobs LC, Markus HS (2017). Vascular risk factor profiles differ between magnetic resonance imaging-defined subtypes of younger-onset lacunar stroke. Stroke.

[10] Ropper AH, Fisher CM, Kleinman GM (1979). Pyramidal infarction in the medulla. A cause of pure motor hemiplegia sparing the face. Neurology.

[11] Etherton MR, Rost NS, Wu O (2018). Infarct topography and functional outcomes. J Cereb Blood Flow Metab.

[12] SatoMD T, SakaiMD K, TakatsuMD H (2020). Infarct site and prognosis in small subcortical infarction: role of the corticospinal tract and lentiform. J Neurol Sci.

[13] Katoh M, Kawamoto T (2000). Bilateral medial medullary infarction. J Clin Neurosci.

[14] Bassetti C, Bogousslavsky J, Mattle H, Bernasconi A (1997). Medial medullary stroke: report of seven patients and review of the literature. Neurology.

[15] Uemura M, Naritomi H, Uno H (2016). Ipsilateral hemiparesis in lateral medullary infarction: clinical investigation of the lesion location on magnetic resonance imaging. J Neurol Sci.

[16] Li X, Wang Y (2014). Lateral medullary infarction with ipsilateral hemiparesis, lemniscal sensation loss and hypoglossal nerve palsy. Neurol Sci.

[17] Saada F, Antonios N (2014). Existence of ipsilateral hemiparesis in ischemic and hemorrhagic stroke: two case reports and review of the literature. Eur Neurol.

[18] Aaron S, Al Hashmi A, Pancharatnam D (2020). Isolated infarction of the medullary pyramid: a rare pure motor stroke. J Stroke Cerebrovasc Dis.

[19] Tan ZR, Zhang C, Tian FF (2019). Spectrum of clinical features and neuroimaging findings in acute cerebral infarction patients with unusual ipsilateral motor impairment- a series of 22 cases. BMC Neurol.

[20] Kataoka S, Hori A, Shirakawa T, Hirose G (1997). Paramedian pontine infarction: neurological/topographical correlation. Stroke.

[21] Porto FH, da Silva SP, Orsini M, de Freitas MR, de Freitas GR (2009). Hemimedullary infarct with ipsilateral hemiplegia: a vertebral artery dissection syndrome?. J Neurol Sci.

[22] Wilkins EG, Kamel H, Johnson EC, Shalev SM, Josephson SA (2012). Ischemic stroke of the pyramidal decussation causing quadriplegia and anarthria. J Stroke Cerebrovasc Dis.

[23] Inatomi Y, Nakajima M, Yonehara T, Ando Y (2017). Ipsilateral hemiparesis in ischemic stroke patients. Acta Neurol Scand.

